# What to do with diabetes therapies when HbA1c lowering is inadequate: add, switch, or continue? A MASTERMIND study

**DOI:** 10.1186/s12916-019-1307-8

**Published:** 2019-04-12

**Authors:** Andrew P. McGovern, John M. Dennis, Beverley M. Shields, Andrew T. Hattersley, Ewan R. Pearson, Angus G. Jones, On behalf of the MASTERMIND Consortium, William E Henley, Mike Lonergan, Lauren R Rodgers, Willie T Hamilton, Naveed A Sattar, Rury R Holman, Catherine Angwin, Kennedy J Cruickshank, Andrew J Farmer, Stephen CL Gough, Alastair M Gray, Christopher Hyde, Christopher Jennison, Mark Walker

**Affiliations:** 1 University of Exeter Medical School, The Institute of Clinical and Biological Sciences, University of Exeter, Exeter, UK; 2 Health Statistics Group, Institute of Health Research, University of Exeter, Exeter, UK; 3 Division of Molecular and Clinical Medicine, Ninewells Hospital and Medical School, University of Dundee, Dundee, UK

**Keywords:** Type 2 diabetes, Oral glucose-lowering medication, HbA1c, Addition, Switching, Continuation, Glycemic control

## Abstract

**Background:**

It is unclear what to do when people with type 2 diabetes have had no or a limited glycemic response to a recently introduced medication. Intra-individual HbA1c variability can obscure true response. Some guidelines suggest stopping apparently ineffective therapy, but no studies have addressed this issue.

**Methods:**

In a retrospective cohort analysis using the UK Clinical Practice Research Datalink (CPRD), we assessed the outcome of 55,530 patients with type 2 diabetes starting their second or third non-insulin glucose-lowering medication, with a baseline HbA1c > 58 mmol/mol (7.5%). For those with no HbA1c improvement or a limited response at 6 months (HbA1c fall < 5.5 mmol/mol [0.5%]), we compared HbA1c 12 months later in those who continued their treatment unchanged, switched to new treatment, or added new treatment.

**Results:**

An increase or a limited reduction in HbA1c was common, occurring in 21.9% (12,168/55,230), who had a mean HbA1c increase of 2.5 mmol/mol (0.2%). After this limited response, continuing therapy was more frequent (*n* = 9308; 74%) than switching (*n* = 1177; 9%) or adding (*n* = 2163; 17%). Twelve months later, in those who switched medication, HbA1c fell (− 6.8 mmol/mol [− 0.6%], 95%CI − 7.7, − 6.0) only slightly more than those who continued unchanged (− 5.1 mmol/mol [− 0.5%], 95%CI − 5.5, − 4.8). Adding another new therapy was associated with a substantially better reduction (− 12.4 mmol/mol [− 1.1%], 95%CI − 13.1, − 11.7). Propensity score-matched subgroups demonstrated similar results.

**Conclusions:**

Where glucose-lowering therapy does not appear effective on initial HbA1c testing, changing agents does not improve glycemic control. The initial agent should be continued with another therapy added.

**Electronic supplementary material:**

The online version of this article (10.1186/s12916-019-1307-8) contains supplementary material, which is available to authorized users.

## Introduction

Current major guidelines for type 2 diabetes suggest checking treatment response to glucose-lowering medication with an HbA1c measurement at 3 to 6 months [1–3]. What is unclear is what to do if there is an apparently poor response; should this treatment be continued with ongoing response monitoring, should it be switched to an alternative medication, or should another medication be added in? To date, no data from randomized controlled trials or observational analyses are available to address this apparently simple but clinically important question. Despite this, national guidance in some regions has recommended discontinuation of therapy if a response threshold on initial HbA1c testing is not met [3].

Data from both trials and observational studies demonstrate that there is a substantial amount of HbA1c variation within individuals on stable therapy over time. This variation produces a standard deviation (SD) of HbA1c within individuals of around 5.0 to 7.1 mmol/mol (0.46 to 0.65%) [4–6]. Assuming an approximately normal distribution of HbA1c variation, one third of people will have an HbA1c variation greater than this SD. As most non-insulin glucose-lowering medications reduce HbA1c by around 11.0 mmol/mol (1.0%), with some variation by class of therapy [7–9], there will be a substantial proportion of people in whom the treatment effect when starting a therapy is at least partially obscured by the biological noise in HbA1c. Given this noise, a reasonable approach to the patient who has apparently had a limited initial HbA1c treatment response might be to continue the therapy unchanged, anticipating that the true response will become apparent over subsequent HbA1c measurements. If this hypothesis is correct, we would expect people who switch to another medication will have similar HbA1c outcomes compared with those continuing the initial therapy unchanged. We would also anticipate people adding another new therapy will experience a greater HbA1c reduction than those who switch despite the apparent lack of effect of the first medication. If the lack of an initial glycemic response is a true reflection of non-response to the new medication, subsequent treatment switching should produce a similar response to adding another medication.

We aimed to establish whether continuing, switching, or adding medication to an apparently ineffective glucose-lowering therapy resulted in the greatest HbA1c improvement.

## Research design and methods

We conducted a retrospective cohort analysis of 55,530 people with type 2 diabetes starting a second or third ever glucose-lowering medication between 2004 and 2017 inclusive. We analyzed those who had either a worsening or a limited improvement in HbA1c (HbA1c fall < 5.5 mmol/mol [0.5%]) 6 months after this additional glucose-lowering therapy. We compared the subsequent glycemic outcomes in those who continued therapy unchanged, switched to an alternative therapy, or added an additional agent.

### Setting and participants

**Fig. 1:**
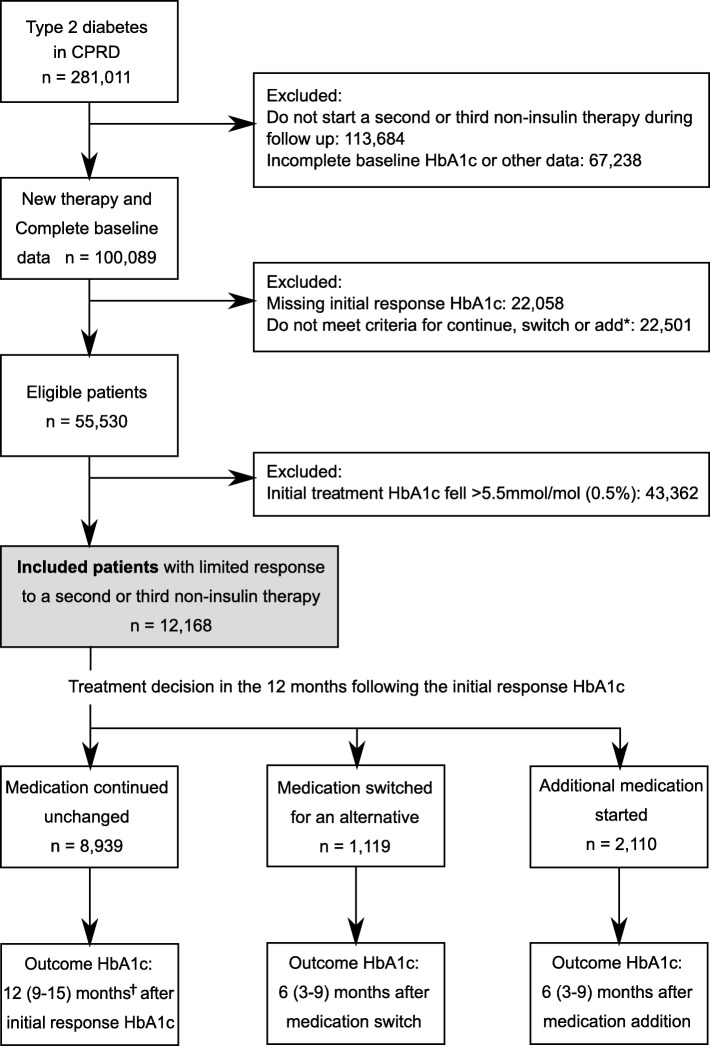
Flow diagram of patient inclusion and follow-up. *Patients failing to meet the criteria for continue, switch, or add had either more complex therapy changes such as adding or switching multiple medications in the 1-year follow-up period, or had reduced adherence to any of the study medications. †This time window was selected to provide an outcome HbA1c measurement in the same time period as the response HbA1c in those switching or adding medications. *CPRD* Clinical Practice Research Datalink

We identified people with type 2 diabetes within the world’s largest longitudinal database of anonymized primary care electronic health records (EHR): the UK Clinical Practice Research Datalink (CPRD) [10]. People with type 2 diabetes were identified as previously described [11]. In brief, people with type 2 diabetes were identified from prescriptions of one or more glucose-lowering therapies in the EHR consistent with a diagnosis of diabetes, and an age of onset of diabetes (first prescription) over 35 years. People were considered not to have type 2 diabetes if they had any diagnosis codes suggesting gestational, genetic, or secondary forms of diabetes, polycystic ovarian syndrome, insulin as their first glucose-lowering medication, or insulin within 1 year of diagnosis. The date of diagnosis of diabetes was defined as the earlier date of the first glucose-lowering medication prescription, first HbA1c value ≥ 48 mmol/mol (6.5%), or first diagnosis code. Where any of these occurred within 3 months of registration with the patients’ practice, the diagnosis date was assumed to predate registration and the person was excluded. We did not put any restrictions on the date of diagnosis. An overview of study design and participant selection is shown in Fig. 1.

### Identification of participants with limited initial HbA1c response to second- and third-line glucose-lowering therapy

We assessed initial HbA1c response in participants commencing second or third glucose-lowering medication (excluding insulin) between 2004 and 2017 (inclusive) meeting the following criteria: new therapy taken continually for at least 6 months (based on prescriptions issued), pretreatment HbA1c (baseline HbA1c) > 58 mmol/mol (7.5%), a measure of post treatment HbA1c after 6 months (initial treatment response HbA1c), ≥ 80% adherence to all prescribed glucose-lowering therapy, and no change in other concurrent glucose-lowering therapy. For the baseline HbA1c, we used the most recent value measured within the 6 months preceding the start of the new medication. We defined the initial treatment response HbA1c as the nearest HbA1c to 6 months (± 3 months) after initiating the new therapy. Non-adherence was defined using medication possession ratio of less than 80% [12–14].

We defined a limited initial response to glucose-lowering therapy as a worsening of HbA1c or an improvement of less than 5.5 mmol/mol (0.5%). We did not examine people with a limited response to a first ever glucose-lowering medication as this is most commonly metformin and the current UK guidelines advocate dose escalation rather than treatment modification where this has occurred [3].

### Participant baseline characteristics

We extracted baseline clinical characteristics at the time of medication start: age, gender, duration of diabetes, and body mass index (BMI), and estimated glomerular filtration rate (eGFR). BMI and eGFR were defined using the most recent record in the 6 months prior to the drug start date.

### Treatment choices and outcomes

From the eligible cohort, three ongoing treatment choices were defined: continue, switch, or add. The continue group was defined as no changes to diabetes therapies for an additional 12 months after the initial treatment response HbA1c. The switch group was defined as people discontinuing their first new therapy and starting an alternative non-insulin glucose-lowering medication within the following 12 months. The add group was defined as continuing their first new therapy and adding a second non-insulin glucose-lowering medication within the following 12 months. In the continue group, the outcome HbA1c was defined as the closest value to 12 months (± 3 months). In the switch and add groups, the outcome HbA1c was defined as the closest value to 6 months (± 3 months) after the medication modification. These time frames were selected to make the follow-up period comparable across the three groups. People without an outcome HbA1c were excluded. People with data fulfilling the inclusion criteria for both their second and third ever medication were only included once, using data for their second medication only.

### Statistical methods

We evaluated differences in baseline characteristics between the continue, switch, and add groups using the *χ*^2^ test for categorical variables and the unpaired *t*-test for continuous data. All reported *p* values are two-sided.

Our primary analysis comprised examination of the HbA1c response to continuing, switching, or adding, in the eligible cohort, with adjustment for baseline variables using linear regression. Linear regression models were adjusted for age, gender, duration of diabetes at the initiation of treatment, baseline HbA1c before the first medication, year of treatment, and line of therapy (second or third ever medication). The response to treatment choice (continue, switch, or add) is reported with 95% confidence intervals (CI) using least-square means. Complete regression model results are also reported. The treatment response was defined as the change in HbA1c between the response HbA1c to their first new therapy (measured at 6 months) and the continue, switch, or add outcome HbA1c (defined for each group as described above).

### Subgroup and sensitivity analyses

We undertook the following subgroup analyses; separate analysis of second- and third-line therapies, a subgroup analysis of those with absolutely no reduction in initial treatment response HbA1c, and subgroups of treatment response to the most commonly modified medication classes. In the latter subgroup analyses, we analyzed the impact of continuing, switching, or adding after a limited response to sulfonylureas, thiazolidinediones (TZDs), and dipeptidyl peptidase-4 (DPP4) inhibitors.

Residual confounding by characteristics influencing treatment decision could influence the results of our primary analysis; this is particularly important for HbA1c, as those with high HbA1c may be more likely to add therapy. To explore this possibility, we performed a sensitivity analysis in propensity score-matched groups. Matching by treatment choice was performed to assess the two most clinically important comparisons: continue compared with switch and switch compared with add. Propensity score matching was performed using a 1:1 ratio and nearest neighbor algorithm. A caliper for matching was set at 0.25 standard deviations [15]. Variables included in the propensity matching were age, gender, duration of diabetes at the initiation of treatment, baseline HbA1c, and HbA1c response to the first therapy (change from the baseline HbA1c to the initial treatment response HbA1c).

A second sensitivity analysis was performed, to explore the possibility of confounding by medication class or line of therapy, using the same group comparisons and matching variables as described above but with the addition of exact matching for first medication and second medication (for comparison of switch and add), and for line of therapy (i.e., if the first medication assessed was the patients’ second ever glucose-lowering medication or their third). An initial caliper for matching of 0.25 standard deviations was not sufficient to remove bias in the propensity score-matched variables following the addition of the exact matching criteria. Therefore, the matching caliper was progressively decreased until there was no significant difference between the matched groups (*p* > 0.05) for baseline HbA1c and HbA1c response to the first therapy. This approach is in accordance with previously published recommendations for caliper selection in 1:1 nearest neighbor propensity score matching where there is a relatively small reservoir of potential matches, whereby the aim is to maximize the group size but minimize the risk of bias [16].

To explore the generalizability of our results, we compared the baseline characteristics of patients who were eligible for inclusion (i.e., those with complete outcome data and where the treatment decision met our definition for continue, switch, or add) with those starting a new second or third medication but excluded (i.e., those with missing outcome data for HbA1c or where the treatment decision was more complex than a simple continuation, switch, or add).

## Results

### A fifth of people starting a second or third therapy had a limited initial HbA1c response

Within CPRD, 55,530 people with type 2 diabetes started a new second or third glucose-lowering medication and had complete baseline and outcome data (Fig. 1). Of these, 21.9% (12,168) had a limited HbA1c response to this new medication in their first 6 months with a mean increase of 2.5 mmol/mol (0.2%). In people with limited response, 8939 (73.5%) continued without changes to medication, 1119 (9.2%) switched medication, and 2110 (17.3%) added a new medication within the first year after the response HbA1c. Those continuing therapy unchanged had older age, longer diabetes duration, lower baseline HbA1c, and a smaller increase in HbA1c at 6 months (Table 1). When medication changes (switch or add) were made, it tended to be early in the 1-year follow-up window. The mean time to outcome HbA1c was therefore slightly longer in the continue (12.2 months; SD 2.1) compared to the switch (10.2 months; SD 4.5; *p* < 0.001) and add groups (10.0 months; SD 4.5; *p* < 0.001).

**Table 1:** The characteristics of people eligible for analysis by treatment choice in the follow-up year

	Continue (*n* = 8939)	Switch (*n* = 1119)	Add (*n* = 2110)	*p*
Age at diagnosis (years)	57.4 (10.5)	54.8 (10.2)	55.3 (10.3)	< 0.001
Female [*n* (%)]	3544 (39.6)	472 (42.2)	865 (41.0)	0.175
BMI (kg/m^2^)	31.5 (6.03)	33.9 (6.6)	33.5 (6.5)	< 0.001
eGFR (ml/min)	77.0 (19.1)	83.4 (17.6)	82.81 (18.3)	< 0.001
Diabetes duration (years)	6.2 (4.7)	5.5 (4.3)	4.9 (4.1)	< 0.001
HbA1c (mmol/mol) before first new medication	69.4 (10.6)	71.9 (11.1)	72.6 (11.8)	< 0.001
First new medication class [*n* (%)]				< 0.001
Metformin	1951 (21.8)	109 (9.7)	302 (14.3)	
Sulfonylureas	2594 (29.0)	203 (18.1)	772 (36.6)	
TZDs	2485 (27.8)	227 (20.3)	335 (15.9)	
Acarbose	127 (1.4)	24 (2.1)	18 (0.9)	
Glinides	101 (1.1)	51 (4.6)	22 (1.0)	
DPP4 inhibitors	1514 (16.9)	447 (39.9)	597 (28.3)	
SGLT2 inhibitors	101 (1.1)	42 (3.8)	40 (1.9)	
GLP1 analogues	66 (0.7)	16 (1.4)	24 (1.1)	
HbA1c (mmol/mol) 6 months after first new medication	71.5 (14.4)	76.2 (15.4)	77.8 (15.4)	< 0.001
Change in HbA1c at 6 months (mmol/mol)*	2.1 (8.3)	4.3 (9.8)	5.2 (10.0)	< 0.001
Second new medication class [*n* (%)]				< 0.001
Metformin	N/A	25 (2.2)	62 (2.9)	
Sulfonylureas	N/A	243 (21.7)	627 (29.7)	
TZDs	N/A	248 (22.2)	621 (29.4)	
Acarbose	N/A	13 (1.2)	57 (2.7)	
Glinides	N/A	34 (3.0)	19 (0.9)	
DPP4 inhibitors	N/A	199 (17.8)	455 (21.6)	
SGLT2 inhibitors	N/A	131 (11.7)	143 (6.8)	
GLP1 analogues	N/A	226 (20.2)	126 (6.0)	

All values are expressed as mean (SD) unless otherwise stated. *A positive change in HbA1c equates to a deterioration. *BMI* body mass index, *eGFR* estimated glomerular filtration rate, *TZD* thiazolidinedione, *DPP4* dipeptidyl peptidase-4, *SGLT2* sodium-glucose co-transporter-2, *GLP1* glucagon-like peptide-1

### Adding therapy led to the greatest improvement in HbA1c

**Fig. 2:**
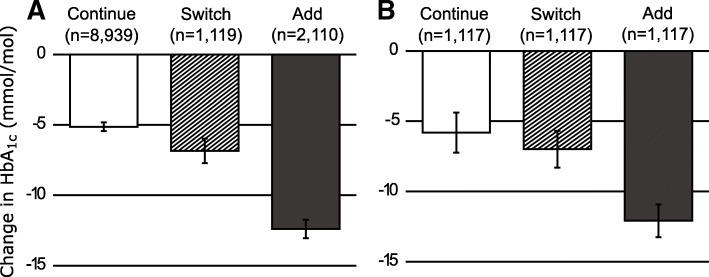
Change in HbA1c 1 year after a limited response to a new medication stratified by treatment decision after response HbA1c: continue, switch, or add. **a** Adjusted HbA1c change in the complete cohort (*n* = 12,168). Response adjusted for age, gender, duration of diabetes, HbA1c at initiation of the first new therapy, year of treatment, and line of therapy (second or third). **b** Unadjusted HbA1c in propensity score-matched groups (*n* = 3351). Matched on age, gender, duration of diabetes, HbA1c at initiation of the first new therapy, and 6-month HbA1c response to the first new therapy

After adjustment for baseline characteristics, the mean HbA1c response to switching was slightly greater than the response to continuing the same therapy: − 6.8 (95%CI − 7.7, − 6.0) mmol/mol (− 0.6%) and − 5.1 (95%CI − 5.5, − 4.8) mmol/mol (− 0.5%) respectively (Fig. 2a); (*p* < 0.001). Those adding additional therapy to an apparently ineffective therapy had the greatest HbA1c reduction: − 12.4 (95%CI − 13.1, − 11.7) mmol/mol (− 1.1%).

### Subgroup and sensitivity analyses

Similar trends were also seen in the subgroups. When analyzed by line of therapy (Additional file 1: Appendix 1) in those who had had a limited response to their second ever glucose-lowering medication, adding was substantially better than continuing (difference − 7.2 mmol/mol [− 0.7%]; 95%CI − 8.0, − 6.3; *p* < 0.001) whereas switching was no better (difference − 0.9 mmol/mol [− 0.1%]; 95%CI − 2.1, 0.3; *p* = 0.127). In those with a limited response to their third ever glucose-lowering medication, adding was also substantially better than continuing (difference − 7.5 mmol/mol [− 0.7%]; 95%CI − 8.8, − 6.1; *p* < 0.001) whereas switching was slightly better (difference − 3.0 mmol/mol [− 0.3%]; 95%CI − 4.4, − 1.6; *p* < 0.001). Restricting the analysis to only those who had an initial worsening of their HbA1c on their new therapy again demonstrated the same trends although the improvement from the 6-month response HbA1c was slightly greater in all groups (Additional file 1: Appendix 2).

Propensity score-matched subgroups demonstrated a similar HbA1c response pattern to the complete cohort analysis (Fig. 2b and Additional file 1: Appendix 3). Baseline characteristics of the comparison groups were well balanced although there were some baseline differences in the medication classes used (Additional file 1: Appendix 3). The result was unchanged when exact matching for line of therapy and medication class was performed (Additional file 1: Appendix 4).

**Fig. 3:**
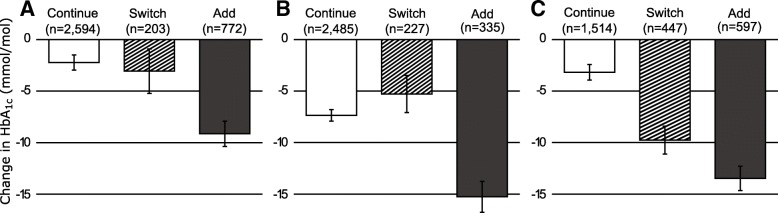
Adjusted change in HbA1c 1 year after a limited response to a sulfonylurea (*n* = 3569) (**a**), thiazolidinedione (*n* = 3047) (**b**), or DPP4 inhibitor (*n* = 2558) (**c**) stratified by the treatment decision after response HbA1c: continue, switch, or add

We found some differences between medication classes (Fig. 3 and Additional file 1: Supplementary Appendix 5): Adding therapy was better than switching or continuing regardless of the original therapy showing the limited response, but the greatest improvements in response were seen when another therapy was added to a TZD. Switching from an SU or TZD did not result in improvements in response compared with continuing the original therapy, but switching from a DPP4 did result in a significant improvement.

Participants eligible for inclusion in the study had similar characteristics to those excluded, with statistically significant but not clinically relevant differences in sex (40.4 vs 42.6% female in eligible vs non eligible; *p* < 0.001), diabetes duration (5.3 vs 5.5 years; *p* < 0.001), and baseline HbA1c (73.4 vs 75.7 mmol/mol; *p* < 0.001) (Additional file 1: Appendix 6).

## Discussion

In our UK-representative cohort, more than a fifth of people with complete data met our criteria for a limited 6-month response to a new therapy, demonstrating that this is common clinical scenario. Of these, the most common practice was to continue the same medication over the following year (73.5%). People continuing and switching did demonstrate a modest improvement in HbA1c over the following year but neither approach improved HbA1c much beyond the baseline HbA1c prior to the first new therapy. Adding another new therapy to the apparently ineffective new therapy was the only approach which led to a clinically significant improvement in HbA1c. These findings were robust across different approaches to analysis. For TZDs, switching was slightly inferior to continuing therapy unchanged, and for DPP4 inhibitors, switching was substantially better than continuing. However, for each of the three classes (sulfonylureas, TZDs, and DPP4 inhibitors), adding another medication lead to the greatest HbA1c reduction, as found with the complete cohort. Our results therefore demonstrate that a single HbA1c measurement cannot be used to identify patients who have limited response to a glucose-lowering therapy. Where a glucose-lowering therapy does not appear to be effective, changing agents does not substantially improve glycemic control. The initial agent should therefore be continued (where that agent is tolerated) and additional therapy added.

The strengths of this study include our use of multiple approaches for exploring the relationship between continuing, switching, and adding therapies, and HbA1c outcomes in a large real-world cohort. We have found the same trends when examined using a complete cohort with results adjusted for baseline characteristics and propensity score-matched groups and in important subgroups.

Limitations of the study include the exclusion of people with incomplete follow-up data; while we cannot assume generalizability to this population, their similar baseline characteristics to those with complete follow-up data is reassuring. We had also insufficient numbers of people treated with some important classes of medication to examine the impact of continuing, switching, or adding at a class level, e.g., sodium glucose co-transporter-2 (SGLT2) inhibitors and glucagon-like peptide-1 (GLP1) analogues. This was predominately because they are newer medication classes and therefore less longitudinal data is currently available. As with all observational studies, we cannot exclude residual confounding; however, we have used multiple methods to attempt to triangulate the impact of treatment choices to reduce this possibility. While we have only included participants who are adherent by medication position ratio in our analysis, this does not fully exclude differences in adherence between treatment groups, as a medicine may be collected and not actually taken. A patient with side effects may be more likely to be non-adherent and subsequently more likely to switch therapy. However, this possibility would not explain the glycemic benefit of adding compared with switching that we identify, or lack of clear benefit from switching in comparison to continuing an “ineffective” therapy. Another limitation of our study was that information on the rationale for treatment decision (continue, switch, or add) was not available from the dataset.

A tendency to continue treatment unchanged in people who have not reached HbA1c targets (termed clinical or treatment inertia) has been reported previously [17–19]. These data are consistent with our finding that continuing a treatment in those with a measured limited HbA1c response was the most common clinical practice. Our results suggest that a large proportion of response variation is due to “noise”—HbA1c variation related to diet or lifestyle change and/or measurement error, rather than innate variation in true “biological” response between individuals. If patients with initial worsening of HbA1c were true “non-responders” to therapy, then switching to a different therapy would be a much more effective strategy than continuing the same ineffective medication. In contrast, our results show little difference between switching therapy and continuing the same medication unchanged—in other words, these patients are obtaining underlying glucose-lowering benefit from the medication, and the initial lack of HbA1c improvement is substantially due to chance variation in HbA1c caused by other, non-treatment-related, factors. If HbA1c is repeated without changing therapy, it will therefore improve due to the effects of regression to the mean [20]. This is entirely consistent with previous studies that show very high variation of HbA1c on stable glucose-lowering therapy [7–9]. This has implications for the study and implementation of stratified or precision approaches to glucose-lowering therapy; while recent studies show it is possible to predict differences in mean glucose-lowering response by patient characteristic or biomarker status [21–24], it may not be possible to predict a person’s response to therapy at an individual level.

Our study is the first to examine the impact of subsequent treatment changes in people with an initially limited treatment response to a new glucose-lowering therapy. A high-quality randomized trial would provide valuable additional data to guide clinic practice in this common scenario and may also allow assessment of whether using multiple HbA1c measures may allow more robust identification of poor responders to a glucose-lowering therapy, something that is not feasible to robustly address in this dataset due to the low frequency of repeat HbA1c measurements in clinical care.

## Conclusion

It is common to have a limited HbA1c response 6 months after starting a new glucose-lowering medication, but this is likely to represent non-treatment-related HbA1c variation, rather than lack of biological response to a therapy. Where a glucose-lowering therapy does not appear to be effective on initial HbA1c testing, changing agents does not improve glycemic control. The initial agent should therefore be continued (where that agent is tolerated) and additional therapy added.

## Additional file

Additional file 1: Supplementary appendices including subgroup analyses, propensity score-matched sensitivity analyses, and a comparison of the characteristics of included and excluded patients. (PDF 156 kb)

## Abbreviations

BMI: Body mass index
CI: Confidence interval
CPRD: Clinical Practice Research Datalink
DPP4: Dipeptidyl peptidase-4
eGFR: Estimated glomerular filtration rate
EHR: Electronic health records
GLP1: Glucagon-like peptide-1
SD: Standard deviation
SGLT2: Sodium glucose co-transporter-2
TZD: Thiazolidinedione
