# Supplementary material for: What to do with diabetes therapies when HbA1c lowering is inadequate: add, switch, or continue? A MASTERMIND study
Source: BMC Med. 2019 Apr 12;17:79. doi: 10.1186/s12916-019-1307-8 (PMC6460517; doi:10.1186/s12916-019-1307-8)
Supplement: Supplementary file 1 — Supplementary appendices including subgroup analyses, propensity score-matched sensitivity analyses, and a comparison of the characteristics of included and excluded patients. (PDF 156 kb) [file 12916_2019_1307_MOESM1_ESM.pdf]

Supplementary Appendices

What to do with diabetes therapies when HbA1c lowering is inadequate: add, switch, or continue? A MASTERMIND study.

Andrew P. McGovern, John M Dennis, Beverley M. Shields, Andrew T. Hattersley, Ewan R. Pearson, Angus G. Jones on behalf of the MASTERMIND consortium

Collaborators in the MASTERMIND consortium

William E Henley (PhD) – University of Exeter, UK
Mike Lonergan (PhD) – University of Dundee, UK
Lauren R Rodgers (PhD) – University of Exeter, UK
Willie T Hamilton (PhD) – University of Exeter, UK
Naveed A Sattar (PhD) – University of Glasgow, UK
Rury R Holman (MD) – University of Oxford, UK
Catherine Angwin (MSc) – University of Exeter, UK
Kennedy J Cruickshank (MD) – Kings College, London, UK
Andrew J Farmer (DM) – University of Oxford, UK
Stephen CL Gough (MD) – University of Oxford, UK
Alastair M Gray (PhD) – University of Oxford, UK
Christopher Hyde (MD) – University of Exeter, UK
Christopher Jennison (PhD) – University of Bath, UK
Mark Walker (MD) – University of Newcastle, UK

Contents

Appendix 1: Subgroup analysis by line of therapy 3

Appendix 2: Subgroup analysis; people with an initial deterioration only 5

Appendix 3: Propensity score matched analysis 6

Appendix 4: Propensity score matched analysis with additional exact matching 8

Appendix 5: Subgroup analysis by medication class 11

Appendix 6: Comparison of the characteristics of included and excluded patients 15

Appendix 1: Subgroup analysis by line of therapy

Subgroup analysis by line of therapy; outcomes after a limited response to a second or third ever glucose lowering medication. A limited response was defined as an HbA$_{1c}$ improvement less than 5.5mmol/mol (including a rise in HbA$_{1c}$) at six months.

	Change in HbA$_{1c}$ (mmol/mol)*		
	2nd & 3rd lines (n=12,168†) *Estimate (95%CI; p)*	2nd line medication (n=7,884) *Estimate (95%CI; p)*	3rd line medication (n=4,764) *Estimate (95%CI; p)*
**Switch**	-1.70 (-2.63, -0.78; p<0.001)	-0.92 (-2.10, 0.26; p=0.127)	-3.00 (-4.39, -1.62; p<0.001)
**Add**	-7.26 (-7.98, -6.54; p<0.001)	-7.17 (-7.99, -6.34; p<0.001)	-7.46 (-8.85, -6.07; p<0.001)
**Age at diagnosis (years)**	-0.11 (-0.13, -0.08; p<0.001)	-0.11 (-0.14, -0.08; p<0.001)	-0.10 (-0.15, -0.06; p<0.001)
**Male**	0.46 (-0.08, 0.99; p=0.094)	0.99 (0.34, 1.65; p=0.003)	-0.57 (-1.43, 0.29; p=0.195)
**Duration of diabetes (years)**	-0.13 (-0.19, -0.07; p<0.001)	-0.16 (-0.24, -0.08; p<0.001)	-0.11 (-0.20, -0.02; p=0.010)
**Year of medication**	0.11 (0.05, 0.16; p<0.001)	0.06 (-0.01, 0.13; p=0.074)	0.23 (0.14, 0.33; p<0.001)
**Baseline HbA$_{1c}$ (mmol/mol)**	-0.43 (-0.46, -0.41; p<0.001)	-0.46 (-0.49, -0.43; p<0.001)	-0.38 (-0.42, -0.34; p<0.001)
**Line of therapy (2nd)**	0.69 (0.11, 1.27; p=0.021)	NA	NA
**Adjusted R^2**	0.106	0.130	0.144

Supplementary Table 1. The impact of switching or adding therapy after an initially limited response to a new medication when compared with continuing the same new therapy unchanged, after adjusting for important covariates. 2nd and 3rd lines of therapy refers to those where their newly initiated medication is their second or third ever glucose lowering medication respectively. *A negative change in HbA$_{1c}$ equates to an improvement. †In the combined analysis of 2nd and 3rd line people with data for both lines were only included once (following their 2nd ever medication). 95%CI = 95% confidence interval.

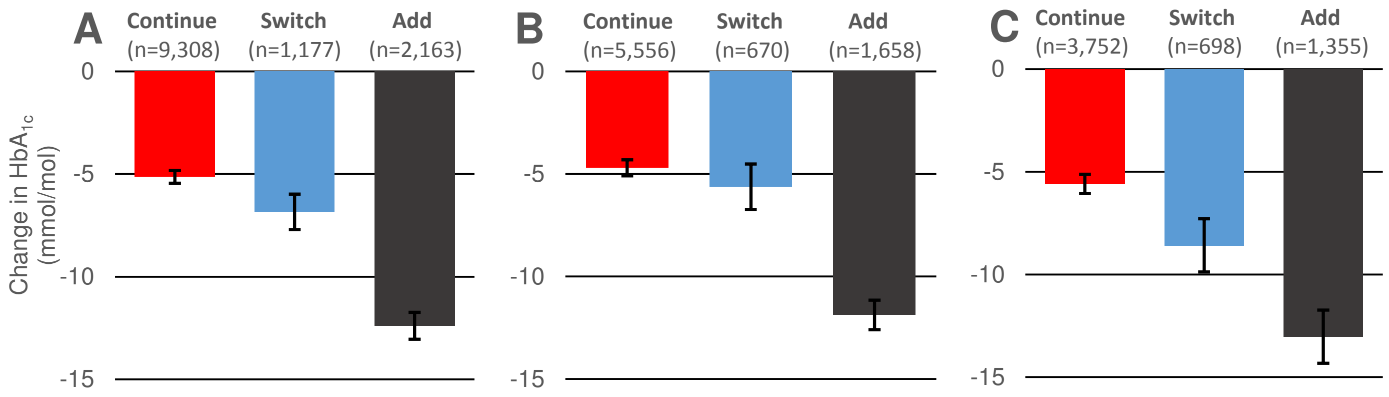

Supplementary Figure 1. The adjusted response in Hb$_{A1c}$ to continuing glucose lowering therapy unchanged, switching, or adding, after an initially limited response to a new medication. **A:** Adjusted changes in the compete cohort (n=12,168) after the initial response HbA1c. **B:** Adjusted changes in those following a limited response to their second ever glucose lowering medication (n=7,884). **C:** Adjusted changes in those following a limited response to their third ever glucose lowering medication (n=4,764).

Appendix 2: Subgroup analysis; people with an initial deterioration only

A subgroup analysis; dataset limited to only those who had an initial rise in HbA_{1c} at six months after starting a new glucose lowering therapy.

	Change in HbA_{1c} (mmol/mol)*		
	2nd & 3rd lines (n=6,197†) *Estimate (95%CI; p)*	2nd line medication (n=3,716) *Estimate (95%CI; p)*	3rd line medication (n=2,481) *Estimate (95%CI; p)*
**Switch**	-1.87 (-3.24, -0.51; p=0.007)	-0.80 (-2.62, 1.02; p=0.391)	-3.41 (-5.47, -1.36; p= 0.001)
**Add**	-6.83 (-7.89, -5.76; p<0.001)	-6.75 (-8.03, -5.48; p<0.001)	-6.59 (-8.55, -4.63; p<0.001)
**Age at diagnosis (years)**	-0.15 (-0.19, -0.11; p<0.001)	-0.16 (-0.21, -0.11; p<0.001)	-0.13 (-0.19, -0.06; p<0.001)
**Male**	0.43 (-0.41, 1.28; p=0.315)	1.13 (0.04, 2.22; p=0.043)	-0.59 (-1.92, 0.75; p=0.390)
**Duration of diabetes (years)**	-0.17 (-0.26, -0.07; p=0.001)	-0.18 (-0.31, -0.05; p=0.009)	-0.14 (-0.28, -0.004; p=0.044)
**Year of medication**	0.12 (0.03, 0.21; p=0.011)	0.06 (-0.05, 0.18; p=0.289)	0.22 (0.06, 0.37; p=0.006)
**Baseline HbA_{1c} (mmol/mol)**	-0.45 (-0.49, -0.42; p<0.001)	-0.51 (-0.55, -0.46; p<0.001)	-0.38 (-0.44, -0.33; p<0.001)
**Line of therapy (2nd)**	0.26 (-0.66, 1.16; p=0.583)	NA	NA
**Adjusted R^2**	0.117	0.090	0.138

Supplementary Table 2. The impact of switching or adding therapy after an initial deterioration in HbA_{1c} to a new medication when compared with continuing the same new therapy unchanged, after adjusting for important covariates. 2nd and 3rd lines of therapy refers to those where their newly initiated medication is their second or third ever glucose lowering medication respectively. *A negative change in HbA_{1c} equates to an improvement. †In the combined analysis of 2nd and 3rd line people with data for both lines were only included once (following their 2nd ever medication) – NB no double inclusions had occurred. 95%CI = 95% confidence interval.

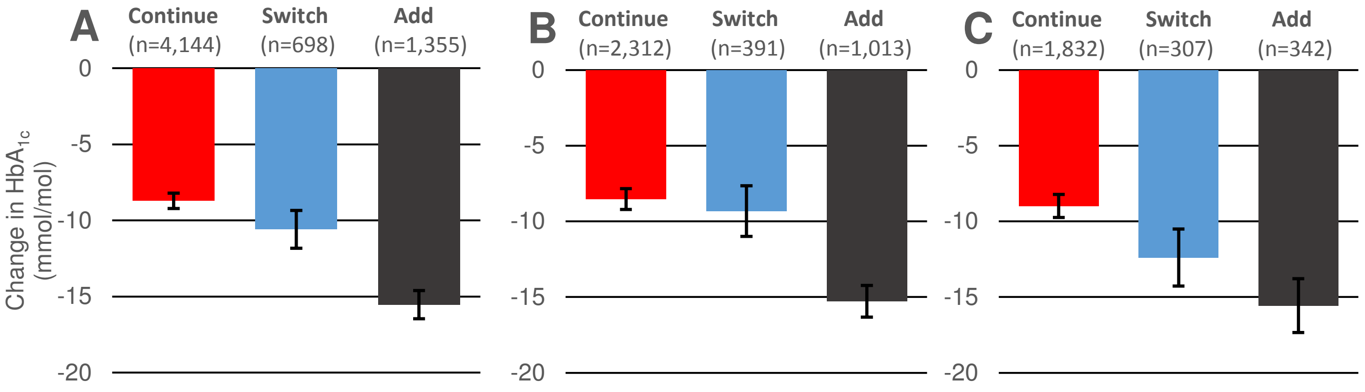

Supplementary Figure 2. The adjusted response in Hb_{A1c} to continuing glucose lowering therapy unchanged, switching, or adding, after an initial deterioration in HbA_{1c} to a new medication. Note the scale change compared to Supplementary figure 1. **A:** Adjusted changes in the compete cohort (n=6,197) after the initial response HbA1c. **B:** Adjusted changes in those following a limited response to their second ever glucose lowering medication (n=3,716). **C:** Adjusted changes in those following a limited response to their third ever glucose lowering medication (n=2,481).

Appendix 3: Propensity score matched analysis

A sensitivity analysis of the impact of continuing, switching, or adding therapy using propensity score matched cohorts. The continue and add groups were 1:1 propensity score matched to the switch group (n=1,119). Two people switching medication could not be matched to the 'add' group and were therefore excluded. Variables included in the propensity matching were age, gender, duration of diabetes at the initiation of treatment, baseline HbA1c, and change in HbA1c at six months (after the first therapy).

	Switch (n=1,117)	Continue (n=1,117)	p	Add (n=1,117)	p
Age at diagnosis (years)	54.8 (10.2)	54.7 (10.1)	0.871	55.1 (10.3)	0.493
Female [n (%)]	472 (42.3)	483 (43.2)	0.669	470 (42.1)	0.966
Diabetes duration (years)	5.5 (4.3)	5.5 (4.2)	0.768	5.3 (4.2)	0.671
Baseline HbA1c (mmol/mol)	71.9 (11.1)	71.9 (12.4)	0.912	72.3 (11.6)	0.355
Change in HbA1c at 6 months (mmol/mol)*	4.2 (9.4)	3.9 (9.9)	0.488	4.2 (8.9)	0.910
First new medication class [n (%)]			<0.001		<0.001
Metformin	109 (9.8)	233 (20.9)		160 (14.3)	
Sulfonylureas	203 (18.2)	360 (32.2)		421 (37.7)	
TZDs	227 (20.3)	288 (25.8)		155 (13.9)	
Acarbose	24 (2.1)	17 (1.5)		11 (1.0)	
Glinides	51 (4.6)	19 (1.7)		12 (1.1)	
DPP4 inhibitors	445 (39.8)	173 (15.5)		314 (28.1)	
SGLT2 inhibitors	42 (3.8)	17 (1.5)		29 (2.6)	
GLP1 analogues	16 (1.4)	10 (0.9)		15 (1.3)	
Second new medication class [n (%)]			NA		<0.001
Metformin	25 (2.2)	-		31 (2.8)	
Sulfonylureas	242 (21.7)	-		324 (29.0)	
TZDs	248 (22.2)	-		335 (30.0)	
Acarbose	13 (1.2)	-		32 (2.9)	
Glinides	34 (3.0)	-		5 (0.4)	
DPP4 inhibitors	199 (17.8)	-		251 (22.5)	
SGLT2 inhibitors	131 (11.7)	-		82 (7.3)	
GLP1 analogues	225 (20.1)	-		57 (5.1)	
Second line therapy [n (%)]	669 (59.9)	691 (61.9)	0.363	864 (77.4)	<0.001

Supplementary Table 3. The characteristics of people included in the propensity score matched sensitivity analyses. All values are expressed as mean (SD) unless otherwise stated. P values provide comparison against the switch group. *A positive change in HbA1c equates to a deterioration. TZD = thiazolidinedione, DPP4 = dipeptidyl peptidase-4, SGLT2 = sodium-glucose co-transporter-2, GLP1 = glucagon-like peptide-1.

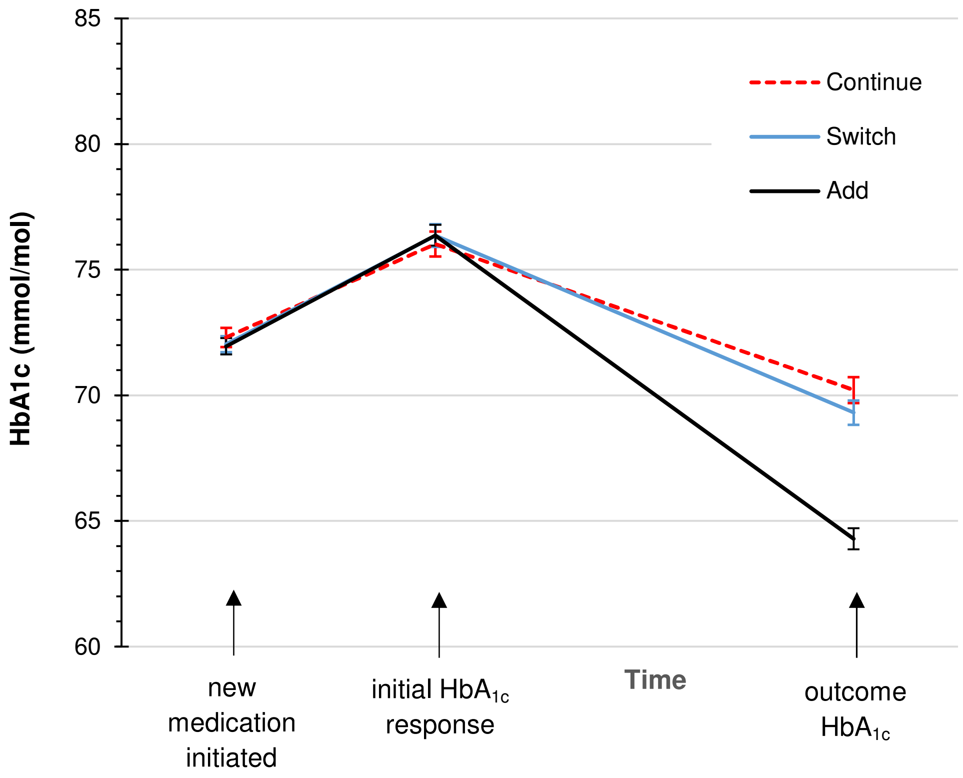

Supplementary Figure 3. Unadjusted Hb_{A1c} changes in response to continuing glucose lowering therapy unchanged (n=1,117), switching (n=1,117), or adding (n=1,117), after an initially limited HbA_{1c} response to a new medication at six months in propensity score matched groups. Note the mean six month response is an increase (deterioration) in HbA_{1c}. The error bars shown represent the standard error of the mean.

Appendix 4: Propensity score matched analysis with additional exact matching

A further sensitivity analysis of the impact of continuing, switching, or adding therapy using propensity score matched cohorts with additional exact matching for line of therapy (2nd or 3rd), initial medication class, and subsequent medication class (when comparing switching and adding). The continue and add groups were 1:1 propensity score matched to the switch group (n=1,117). Variables included in the propensity matching were same as those used for the analysis in Appendix 3 with the addition of the exact matching variables.

	**Continue** (n=803)	**Switch** (n=803)	**p**
**Age at diagnosis (years)**	55.3 (10.0)	56.3 (10.0)	0.058
**Female [n (%)]**	308 (38.4)	313 (39.0)	0.838
**Diabetes duration (years)**	5.5 (4.2)	5.8 (4.2)	0.301
**Baseline HbA1c (mmol/mol)**	69.3 (10.0)	70.4 (10.0)	0.035
**Change in HbA1c at 6 months (mmol/mol)***	2.00 (7.6)	2.7 (7.6)	0.064
**First new medication class [n (%)]**			1.000
**Metformin**	99 (12.3)	99 (12.3)	
**Sulfonylureas**	178 (22.2)	178 (22.2)	
**TZDs**	198 (24.7)	198 (24.7)	
**Acarbose**	5 (0.6)	5 (0.6)	
**Glinides**	6 (0.7)	6 (0.7)	
**DPP4 inhibitors**	308 (38.4)	308 (38.4)	
**SGLT2 inhibitors**	6 (0.7)	6 (0.7)	
**GLP1 analogues**	3 (0.4)	3 (0.4)	
**Second new medication class [n (%)]**			NA
**Metformin**	-	10 (1.2)	
**Sulfonylureas**	-	163 (20.3)	
**TZDs**	-	186 (23.2)	
**Acarbose**	-	11 (1.4)	
**Glinides**	-	32 (4.0)	
**DPP4 inhibitors**	-	154 (19.2)	
**SGLT2 inhibitors**	-	93 (11.6)	
**GLP1 analogues**	-	154 (19.2)	
**Second line therapy [n (%)]**	484 (60.3)	484 (60.3)	1.000

Supplementary Table 4. The characteristics of people included in the matched analysis, comparing people switching therapy to those continuing unchanged, using both propensity score and exact matching. All values are expressed as mean (SD) unless otherwise stated. P values provide comparison against the switch group. *A positive change in HbA1c equates to a deterioration. TZD = thiazolidinedione, DPP4 = dipeptidyl peptidase-4, SGLT2 = sodium-glucose co-transporter-2, GLP1 = glucagon-like peptide-1.

	**Switch** (n=229)	**Add** (n=229)	p
Age at diagnosis (years)	57.2 (9.6)	55.3 (10.0)	0.045
Female [n (%)]	99 (43.2)	89 (38.9)	0.393
Diabetes duration (years)	4.0 (3.0)	4.0 (3.1)	0.909
Baseline HbA1c (mmol/mol)	70.6 (10.6)	70.6 (10.0)	0.950
Change in HbA1c at 6 months (mmol/mol)*	2.7 (7.3)	3.4 (7.3)	0.289
First new medication class [n (%)]			1.000
Metformin	32 (14.0)	32 (14.0)	
Sulfonylureas	84 (36.7)	84 (36.7)	
TZDs	36 (15.7)	36 (15.7)	
Acarbose	1 (0.4)	1 (0.4)	
Glinides	0 (0.0)	0 (0.0)	
DPP4 inhibitors	74 (32.3)	74 (32.3)	
SGLT2 inhibitors	2 (0.9)	2 (0.9)	
GLP1 analogues	0 (0.0)	0 (0.0)	
Second new medication class [n (%)]			1.000
Metformin	3 (1.3)	3 (1.3)	
Sulfonylureas	81 (35.4)	81 (35.4)	
TZDs	76 (33.2)	76 (33.2)	
Acarbose	0 (0.0)	0 (0.0)	
Glinides	1 (0.4)	1 (0.4)	
DPP4 inhibitors	45 (19.7)	45 (19.7)	
SGLT2 inhibitors	15 (6.6)	15 (6.6)	
GLP1 analogues	8 (3.5)	8 (3.5)	
Second line therapy [n (%)]	198 (86.5)	198 (86.5)	1.000

Supplementary Table 5. The characteristics of people included in the matched analysis, comparing people switching therapy to those adding, using both propensity score and exact matching. All values are expressed as mean (SD) unless otherwise stated. P values provide comparison against the switch group. *A positive change in HbA1c equates to a deterioration. TZD = thiazolidinedione, DPP4 = dipeptidyl peptidase-4, SGLT2 = sodium-glucose co-transporter-2, GLP1 = glucagon-like peptide-1.

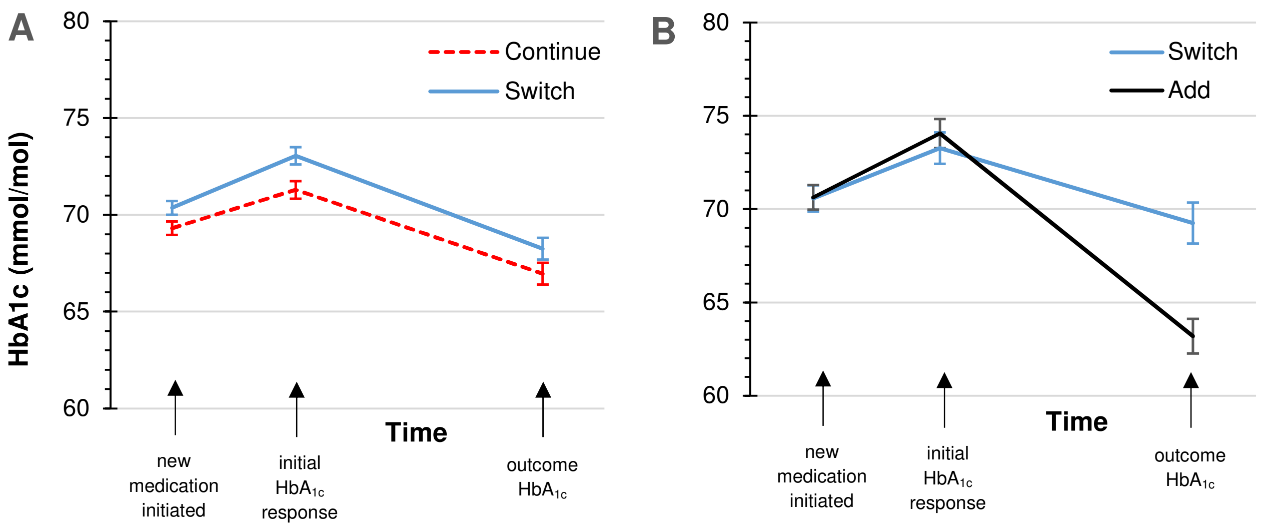

Supplementary Figure 4. Unadjusted Hb_{A1c} changes in response to continuing glucose lowering therapy unchanged, switching, or adding, after an initially limited HbA_{1c} response to a new medication at six months in groups matched using exact and propensity score matching. Note the mean six month response is an increase (deterioration) in HbA_{1c}. The error bars shown represent the standard error of the mean. **A:** Matched continue (n=803) and switch (n=803) groups (characteristics in Supplementary Table 4). **B:** Matched switch (n=229) and add (n=229) groups (characteristics in Supplementary Table 5).

Appendix 5: Subgroup analysis by medication class

A subgroup analysis of the impact of continuing, switching, or adding therapy in people who have had a limited response to a sulfonylurea (n=3,569), thiazolidinedione (n=3,047), or DPP4 inhibitor (n=2,558).

	**Continue** (n=2,594)	**Switch** (n=203)	**Add** (n=772)	**p**
**Age at diagnosis (years)**	58.0 (10.9)	55.0 (10.5)	56.2 (10.3)	<0.001
**Female [n (%)]**	1250 (48.2)	104 (51.2)	383 (49.6)	0.592
**Diabetes duration (years)**	4.7 (3.6)	4.3 (3.5)	4.2 (3.5)	0.001
**Baseline HbA1c (mmol/mol)**	68.7 (10.1)	69.7 (10.3)	72.4 (11.1)	<0.001
**Change in HbA1c at 6 months (mmol/mol)***	2.0 (8.3)	3.5 (9.3)	5.0 (9.9)	<0.001
**First new medication class [n (%)]**				
**Sulfonylureas**	2,594 (100.0)	203 (100.0)	772 (100.0)	
**Second new medication class [n (%)]**				
**Metformin**	-	0 (0.0)	6 (0.8)	
**Sulfonylureas**	-	-	-	
**TZDs**	-	0 (0.0)	15 (1.9)	
**Acarbose**	-	8 (3.9)	1 (0.1)	
**Glinides**	-	86 (42.4)	332 (43.0)	
**DPP4 inhibitors**	-	9 (4.4)	26 (3.4)	
**SGLT2 inhibitors**	-	25 (12.3)	71 (9.2)	
**GLP1 analogues**	-	0 (0.0)	15 (1.9)	
**Second line therapy [n (%)]**	2,126 (82.0)	166 (81.8)	676 (87.6)	0.001

Supplementary Table 6. The characteristics of people included in the subgroup comparing outcomes after a limited response to a sulfonylurea. All values are expressed as mean (SD) unless otherwise stated. P values provide comparison against the switch group. *A positive change in HbA1c equates to a deterioration. TZD = thiazolidinedione, DPP4 = dipeptidyl peptidase-4, SGLT2 = sodium-glucose co-transporter-2, GLP1 = glucagon-like peptide-1.

	**Continue** (n=2,485)	**Switch** (n=227)	**Add** (n=335)	**p**
**Age at diagnosis (years)**	56.1 (9.8)	55.3 (10.7)	54.7 (10.8)	0.035
**Female [n (%)]**	836 (33.6)	80 (35.2)	98 (29.3)	0.225
**Diabetes duration (years)**	6.7 (5.0)	5.4 (4.0)	4.8 (4.2)	<0.001
**Baseline HbA1c (mmol/mol)**	69.3 (10.5)	71.1 (10.4)	73.4 (12.8)	<0.001
**Change in HbA1c at 6 months (mmol/mol)***	2.4 (8.8)	3.9 (8.5)	4.4 (8.5)	<0.001
**First new medication class [n (%)]**				
**TZDs**	2,485 (100.0)	227 (100.0)	335 (100.0)	
**Second new medication class [n (%)]**				
**Metformin**	-	11 (4.8)	34 (10.1)	
**Sulfonylureas**	-	91 (40.1)	207 (61.8)	
**TZDs**	-	-	-	
**Acarbose**	-	8 (3.5)	21 (6.3)	
**Glinides**	-	15 (6.6)	5 (1.5)	
**DPP4 inhibitors**	-	70 (30.8)	52 (15.5)	
**SGLT2 inhibitors**	-	9 (4.0)	2 (0.6)	
**GLP1 analogues**	-	23 (10.1)	14 (4.2)	
**Second line therapy [n (%)]**	945 (38.0)	125 (55.1)	261 (77.9)	<0.001

Supplementary Table 7. The characteristics of people included in the subgroup comparing outcomes after a limited response to a thiazolidinedione. All values are expressed as mean (SD) unless otherwise stated. P values provide comparison against the switch group. *A positive change in HbA1c equates to a deterioration. TZD = thiazolidinedione, DPP4 = dipeptidyl peptidase-4, SGLT2 = sodium-glucose co-transporter-2, GLP1 = glucagon-like peptide-1.

	**Continue** (n=1,514)	**Switch** (n=447)	**Add** (n=597)	**p**
**Age at diagnosis (years)**	56.9 (10.2)	53.6 (9.1)	54.5 (10.0)	<0.001
**Female [n (%)]**	561 (37.1)	181 (40.5)	231 (38.7)	0.392
**Diabetes duration (years)**	7.1 (4.7)	6.0 (4.5)	5.4 (4.1)	<0.001
**Baseline HbA1c (mmol/mol)**	68.7 (10.4)	72.4 (11.2)	71.9 (11.9)	<0.001
**Change in HbA1c at 6 months (mmol/mol)***	1.3 (7.9)	5.3 (11.4)	5.5 (10.5)	<0.001
**First new medication class [n (%)]**				
**DPP4 inhibitors**	1,514 (100.0)	447 (100.0)	597 (100.0)	
**Second new medication class [n (%)]**				
**Metformin**	-	5 (1.1)	11 (1.8)	
**Sulfonylureas**	-	109 (24.4)	364 (61.0)	
**TZDs**	-	58 (13.0)	78 (13.1)	
**Acarbose**	-	1 (0.2)	2 (0.3)	
**Glinides**	-	2 (0.4)	2 (0.3)	
**DPP4 inhibitors**	-	-	-	
**SGLT2 inhibitors**	-	104 (23.3)	107 (17.9)	
**GLP1 analogues**	-	168 (37.6)	33 (5.5)	
**Second line therapy [n (%)]**	568 (37.5)	215 (48.1)	395 (66.2)	<0.001

Supplementary Table 8. The characteristics of people included in the subgroup comparing outcomes after a limited response to a DPP4 inhibitor. All values are expressed as mean (SD) unless otherwise stated. P values provide comparison against the switch group. *A positive change in HbA1c equates to a deterioration. TZD = thiazolidinedione, DPP4 = dipeptidyl peptidase-4, SGLT2 = sodium-glucose co-transporter-2, GLP1 = glucagon-like peptide-1.

	Change in HbA_{1c} (mmol/mol)*		
	**Sulfonylureas (n=3,569)** *Estimate (95%CI; p)*	**TZDs (n=3,047)** *Estimate (95%CI; p)*	**DPP4 inhibitors (n=2,558)** *Estimate (95%CI; p)*
**Switch**	-0.84 (-3.03, 1.36; p=0.455)	2.06 (0.17, 3.96; p=0.033)	-6.58 (-8.14, -5.02; p<0.001)
**Add**	-6.91 (-8.17, -5.66; p<0.001)	-7.91 (-9.55, -6.27; p<0.001)	-10.30 (-11.74, -8.87; p<0.001)
**Age at diagnosis (years)**	-0.10 (-0.14, -0.05; p<0.001)	-0.09 (-0.14, -0.04; p<0.001)	-0.11 (-0.17, -0.06; p<0.001)
**Male**	0.34 (-0.67, 1.35; p=0.513)	-1.54 (-2.57, -0.51; p=0.004)	0.61 (-0.54, 1.77; p=0.299)
**Duration of diabetes (years)**	-0.12 (-0.27, 0.03; p=0.107)	0.02 (-0.09, 0.13; p=0.689)	-0.15 (-0.28, -0.02; p=0.029)
**Year of medication**	0.00 (-0.12, 0.12; p=0.960)	0.16 (-0.01, 0.33; p=0.072)	-0.05 (-0.31, 0.22; p=0.737)
**Baseline HbA_{1c} (mmol/mol)**	-0.47 (-0.52, -0.42; p<0.001)	-0.43 (-0.47, -0.38; p<0.001)	-0.39 (-0.44, -0.34; p<0.001)
**Line of therapy (2nd)**	0.61 (-0.78, 2.00; p=0.392)	0.87 (-0.21, 1.95; p=0.116)	0.05 (-1.19, 1.29; p=0.943)
**Adjusted R^2**	0.117	0.090	0.138

Supplementary Table 9. The impact of switching or adding therapy after an initial deterioration in HbA_{1c} on sulfonylureas, TZDs, and DPP4 inhibitors, when compared with continuing the same new therapy unchanged, after adjusting for important covariates. *A positive change in HbA1c equates to a deterioration. 95%CI = 95% confidence interval, TZD = thiazolidinedione, DPP4 = dipeptidyl peptidase-4.

Appendix 6: Comparison of the characteristics of included and excluded patients

To explore the generalisability of our results we compared the characteristics patients who were eligible for inclusion (i.e. those with complete outcome data and where the treatment decision met our definition for continue, switch, or add) and those starting a new second or third medication but excluded (i.e. those with missing outcome data for HbA1c or where the treatment decision was more complex than a simple continuation, switch, or add). For this analysis we compare all those potentially eligible for inclusion (not just those with a limited treatment response).

	**Eligible** (n=55,530)	**Excluded** (n=44,559)	**p**
**Age at diagnosis (years)**	58.5 (10.6)	58.3 (11.4)	0.058
**Female [n (%)]**	22,449 (40.4)	18,991 (42.6)	<0.001
**BMI (kg/m^2)**	31.7 (6.2)	31.8 (6.5)	0.054
**eGFR (ml/min)**	76.6 (19.6)	76.4 (21.1)	0.330
**Diabetes duration (years)**	5.3 (4.6)	5.5 (4.9)	<0.001
**Baseline HbA1c (mmol/mol)**	73.4 (17.2)	75.7 (19.3)	<0.001
**First new medication class [n (%)]**			<0.001
**Metformin**	11,614 (20.9)	8,441 (18.9)	
**Sulfonylureas**	22,795 (41.0)	17,597 (39.5)	
**TZDs**	11,509 (20.7)	8,065 (18.1)	
**Acarbose**	478 (0.9)	565 (1.3)	
**Glinides**	504 (0.9)	391 (0.9)	
**DPP4 inhibitors**	7,520 (13.5)	7,625 (17.1)	
**SGLT2 inhibitors**	551 (1.0)	1131 (2.5)	
**GLP1 analogues**	559 (1.0)	744 (1.7)	
**Second line therapy [n (%)]**	44,123 (79.5)	33,485 (75.1)	<0.001

Supplementary Table 10. A comparison of the characteristics of people eligible for inclusion with those excluded from inclusion. All values are expressed as mean (SD) unless otherwise stated. TZD = thiazolidinedione, DPP4 = dipeptidyl peptidase-4, SGLT2 = sodium-glucose co-transporter-2, GLP1 = glucagon-like peptide-1.
